# Ectoparasite Diversity and Infection Burden on Two Sympatric Bat Species, *Myotis lucifugus* and *M. septentrionalis* (Chiroptera: Vespertilionidae)

**DOI:** 10.1002/ece3.72602

**Published:** 2025-12-02

**Authors:** Alexandra H. Sauk, Hugh G. Broders

**Affiliations:** ^1^ Department of Biology University of Waterloo Waterloo Ontario Canada

**Keywords:** host–parasite dynamics, Ischnopsyllidae, parasite ecology, social behavior, Spinturnicidae

## Abstract

Parasites are an abundant and diverse group of organisms that are often excluded from biodiversity surveys, limiting our understanding of host–parasite relationships and parasite diversity. Parasites are dependent on their hosts for survival and parasite populations are at risk if their host populations decline. The aim of our study was to quantify and compare the ectoparasite communities of two sympatric Atlantic Canadian bat species, *Myotis lucifugus* and *M. septentrionalis* . Ectoparasites were collected from bats captured for research throughout Atlantic Canada between 1999 and 2017 during the active season (May–October). The prevalence and mean intensity of infection were calculated for each identified ectoparasite species and generalized linear mixed models were used to assess host differences in infection by the two most abundant ectoparasites. Both bat species hosted ectoparasite communities dominated by the mite *Spinturnix americanus* and the flea *Myodopsylla insignis* with other ectoparasites being rarely encountered. Despite being the most common ectoparasites of both bat species, our results suggest that infections of these ectoparasites vary between them with *M. insignis* prevalence being greater on *M. lucifugus* and *S. americanus* prevalence being greater on *M. septentrionalis.* We suggest these differences in infection burden are related to the social and roosting behaviors of these hosts and the life history of the ectoparasites. Monitoring parasites concurrently with focal species is important for capturing this aspect of biodiversity and for understanding how host–parasite dynamics may be disrupted if hosts undergo drastic demographic changes.

## Graphical abstract

Ectoparasite community diversity is similar between two endangered bat species in Atlantic Canada. Ectoparasite burden of the two most common ectoparasites varies between bat species in conjunction with their behavior and roosting differences.

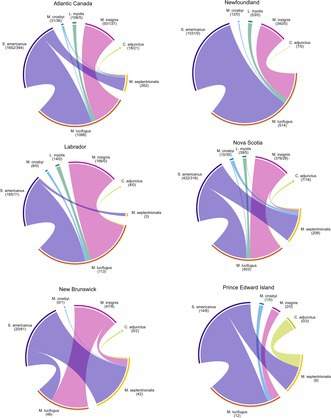

## 1 Introduction

Parasites are an evolutionarily successful and diverse group of organisms estimated to account for more than half of extant animal species (Bordes and Morand 2015; Krasnov and Poulin 2015). Despite this diversity, parasites are often excluded or missed from ecological surveys for the very reasons that make them effective: they are small, hidden in or on a host, and have patchy distributions (Galloway and Danks 1991; Gómez and Nichols 2013; Okamura et al. 2018). The paucity of data on parasites means that biodiversity estimates from some ecological surveys may be under representations of actual biodiversity (Galloway and Danks 1991; Gómez and Nichols 2013; Bennett et al. 2021). This under representation limits our knowledge of parasite communities and host–parasite relationships, leading to bias toward larger metazoan parasites, particularly those of medical, veterinary, and agricultural importance (Bordes and Morand 2015; Okamura et al. 2018). Additionally, understanding what factors influence parasite diversity and communities is essential for understanding the evolution of parasites and the dynamics of host–parasite relationships.

Host characteristics such as size, sex, and immunocompetence can influence the diversity and abundance of parasites infecting a host (Poulin and Valtonen 2001; Cox and John‐Alder 2007; Duneau and Ebert 2012; Waterman et al. 2014; Fagir et al. 2015; Webber, Czenze, and Willis 2015). Behavioral differences between hosts may affect the encounter or transmission rates of parasites. For example, males of many rodent species have greater home ranges than females and travel over larger distances, facilitating interaction with more parasites (Krasnov et al. 2011; Kowalski et al. 2015). Differences in sociality between sexes (Waterman et al. 2014) or differences in foraging locations (Reimchen and Nosil 2001) may also explain variation in parasite community composition. Variation in host behavior in conjunction with environmental factors may also affect ectoparasites as host individuals can choose different nesting and foraging locations with varying microclimates or ecological characteristics (Gómez‐Díaz et al. 2008; Mize et al. 2011; Castaño‐Vázquez et al. 2022). Temperature and humidity affect when ectoparasite reproduction and development occur, leading to seasonal or regional variation in ectoparasite abundance and burden on hosts (Marshall 1982; Benoit and Denlinger 2010; Yaro et al. 2012). The variation among hosts and environments shapes ectoparasite communities and contributes to the aggregated distributions characteristic of parasitic species (Shaw and Dobson 1995; Poulin 2007).

One host group with great species diversity and variation in host characteristics and behaviors within and between species is bats (Order: Chiroptera). Ectoparasite assemblages of bats often consist of both insect and acarine ectoparasites and can vary between bat species and by location. For example, big brown bats ( *Eptesicus fuscus* ) in Colorado and little brown myotis ( *Myotis lucifugus* ) in Manitoba have similar group‐living behaviors but host different numbers of ectoparasite species (11 species and two species, respectively; Pearce and O'Shea 2007; Webber, McGuire, et al. 2015). The different roosting behaviors of bats, including cave‐dwelling (Niu et al. 2007; Deleva and Chaverri 2018), tree‐roosting (Vonhof and Barclay 1996; Boyles and Aubrey 2006; Kühnert et al. 2016), and living in anthropogenic structures (Pearce and O'Shea 2007; Postawa et al. 2014; Kühnert et al. 2016), may impose different environmental selection pressures on ectoparasite communities (Poissant et al. 2010; Postawa et al. 2014). Additionally, social organization differs between bat species and may influence the rate of ectoparasite transmission between individuals and sexes (Czenze and Broders 2011; Presley 2012; Webber, McGuire, et al. 2015).

The little brown myotis ( *Myotis lucifugus* ) and northern myotis ( *Myotis septentrionalis* ) are sympatric species of Vespertilionid bats with large geographic ranges in North America (Fenton and Barclay 1980; Caceres and Barclay 2000). Both species are insectivorous and of similar sizes (Fenton and Barclay 1980; Caceres and Barclay 2000). Females of both species form maternity colonies during the summer months with males roosting individually or in small groups (Davis and Hitchcock 1959; Fenton and Barclay 1980; Anthony et al. 1981; Foster and Kurta 1999; Broders and Forbes 2004; Lacki et al. 2009). The roosting behaviors vary between the two species with *M. lucifugus* congregating in larger roost groups with high roost fidelity for a select number of preferred roosts and the smaller groups of *M. septentrionalis* switching to new roosts more frequently (Lewis 1995; Menzel et al. 2002; Broders and Forbes 2004; Johnson et al. 2009). Both species of bats roost in trees; however, *M. lucifugus* appears to have a much greater affinity for anthropogenic structures, including houses and bat boxes (Davis and Hitchcock 1959; Fenton and Barclay 1980; Foster and Kurta 1999; Broders and Forbes 2004; Lacki et al. 2009; Randall et al. 2014).

This study aimed to quantify and compare the diversity of ectoparasites infecting *M. lucifugus* and *M. septentrionalis* across Atlantic Canada and assess patterns of infection across host and ectoparasite species. This study builds on the previous work by Czenze and Broders (2011) that characterized bat ectoparasite assemblages of *M. lucifugus* and *M. septentrionalis* at fall swarming sites in Nova Scotia and New Brunswick and expands it across Atlantic Canada for both summer maternity and fall swarming sites. Both *M. lucifugus* and *M. septentrionalis* were found with infections of *Spinturnix americanus* (Order: Mesostigmata), *Macronyssus crosbyi* (Order: Mesostigmata), and *Myodopsylla insignis* (Order: Siphonaptera) in Nova Scotia and New Brunswick, with *M. septentrionalis* hosting an additional three ectoparasite species (Czenze and Broders 2011). Both bat species were also infected with *Leptotrombidium myotis* (Order: Trombidiformes) at Hayes Cave in Nova Scotia (Poissant and Broders 2008).

Hosts with shared evolutionary history are more likely to have parasite species in common from a shared ancestor or possess similar traits that make them susceptible to the same parasites (Poulin 2007), As such we expected *M. lucifugus* and *M. septentrionalis* would have a similar diversity of ectoparasites. *Myotis lucifugus* and *M. septentrionalis* are sympatric species within the monophyletic Nearctic New World Myotis clade whose shared common ancestor was 6–8.5 million years ago (Stadelmann et al. 2007). In contrast, we hypothesized that differences in roosting dynamics and aggregation sizes would affect the transmission of ectoparasites and the maintenance of infections. We predicted that *M. lucifugus* would have higher infection prevalence (percent of examined bats that are infected) and intensity (number of ectoparasite individuals per examined bat) than *M. septentrionalis* due to their larger aggregations and higher roost fidelity. Prevalence and mean intensity of infection for both host species were assessed across their range in Atlantic Canada and related to the biology of the hosts and ectoparasites.

## 2 Materials and Methods

### 2.1 Study Area

**FIGURE 1:**
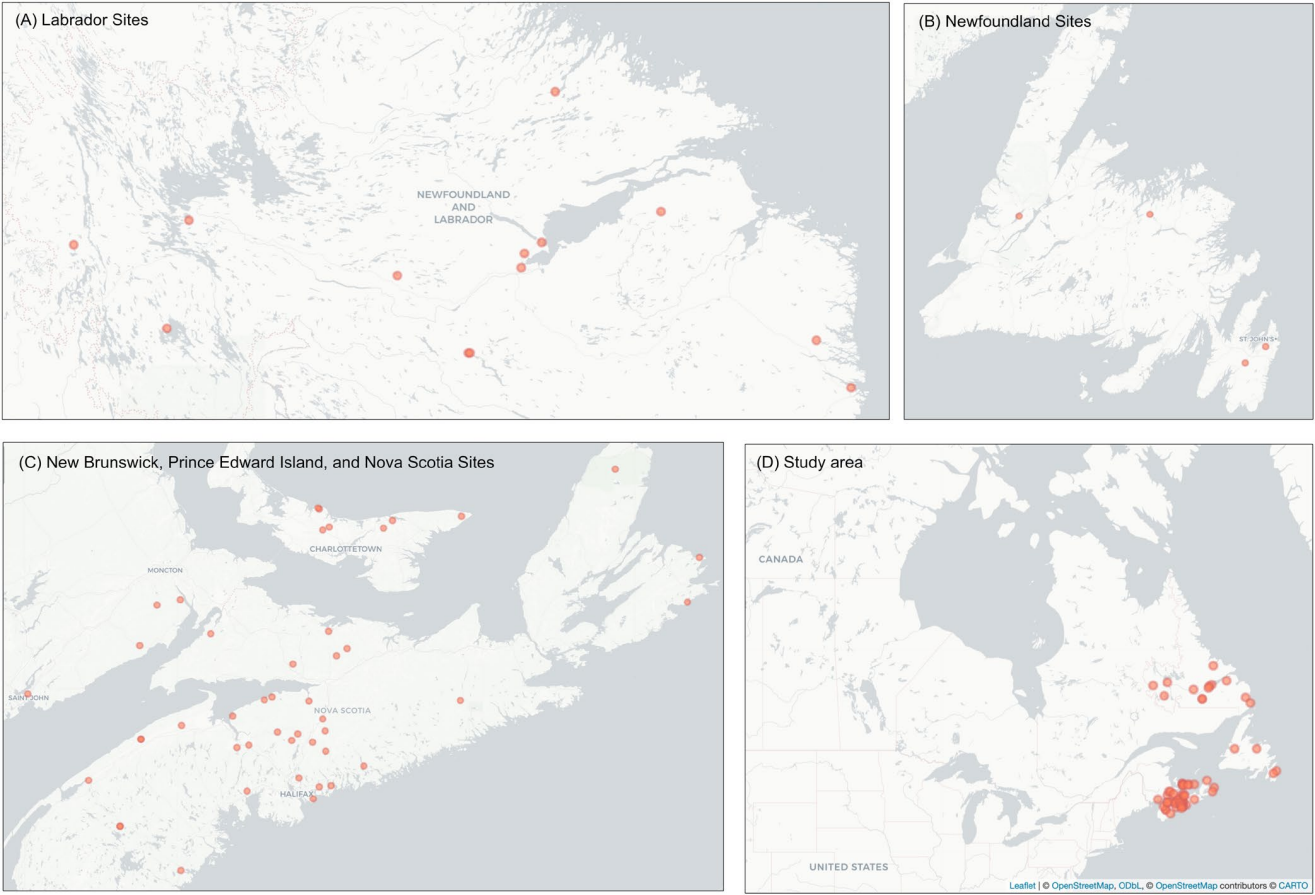
Map of ectoparasite collection locations in Atlantic Canada. A zoomed‐in view is presented for locations in (A) Labrador, (B) the island of Newfoundland, and (C) New Brunswick, Prince Edward Island, and Nova Scotia. View (D) shows the location of the study area within North America. Map visualized using the package *leaflet* in R with map tiles by CARTO (CC BY 3.0) and map data from OpenStreetMap (openstreetmap.org/copyright).

The ectoparasites in this study were collected as part of long‐term monitoring and other research projects of little brown myotis ( *Myotis lucifugus* ) and northern myotis ( *M. septentrionalis* ) in Atlantic Canada (for examples, see Poissant and Broders 2008; Poissant et al. 2010; Czenze and Broders 2011; Burns and Broders 2014, 2015a, 2015b; Burns et al. 2014; McLeod et al. 2015; Sunga et al. 2024). Bat captures were carried out at both maternity sites, where female bats congregate to have and raise their young during the spring and summer months, and swarming sites where bats from multiple maternity sites and male bats congregate prior to hibernation (Davis and Hitchcock 1959; Fenton and Barclay 1980; Anthony et al. 1981; Foster and Kurta 1999; Broders and Forbes 2004; Lacki et al. 2009). Locations include anthropogenic structures (e.g., houses, bat boxes, and mine shafts), natural caves, flyways within forests and near waterways. The ectoparasites were collected between 1999 and 2017 from 60 sites across Nova Scotia, New Brunswick, Prince Edward Island, and Newfoundland and Labrador (Figure 1). Collections occurred between May and October depending on the study period of the individual projects. Collection metadata, including collection dates for each ectoparasite, is available in the Dryad data repository (DOI: https://doi.org/10.5061/dryad.h9w0vt4v3).

### 2.2 Ectoparasite Collection and Identification

Bats were caught in mist nets (Avinet, Dryden, New York, USA) and/or harp traps (Austbat Research Equipment, Lower Plenty, Victoria, Australia) and examined visually to determine their species, sex, and age. Standard bat handling procedures require the wings be opened to measure forearm length and to visually assess the age of the bat by examining the ossification of the metacarpal‐phalangeal joint. During this process, visible ectoparasites were collected from the wings, body and ears of the bats when time and resources permitted with priority given to minimizing handling time and stress for the bats. Some systematic ectoparasite sampling was completed for specific projects (see Poissant and Broders 2008; Czenze and Broders 2011). Bats that were not examined for ectoparasites were not included as part of this study as the presence or absence of ectoparasites could not be confirmed. Ectoparasites were removed using forceps and stored in 70% ethanol and frozen at −20°C or −80°C for long‐term storage. Ectoparasites collected and/or identified during previous work by Czenze and Broders (2011), Talbot et al. (2016), and Banerjee et al. (2020) are included as part of the larger collection.

Ectoparasites were examined using microscopy and identified to the lowest taxonomic rank possible using morphological characteristics. *Spinturnix americanus* is one of several mites found on bats in Atlantic Canada (Poissant and Broders 2008; Czenze and Broders 2011). *Spinturnix americanus* can be distinguished by the four pairs of legs that are large in relation to their body size and organized radially around the idiosoma (Whitaker 1982). In comparison, chiggers (Trombiculidae) are only parasitic during their larval stages, possessing only three pairs of legs which are much smaller in relation to their bodies and positioned anteriorly (Ebeling 1975; Whitaker 1982). *Spinturnix americanus* is similar in appearance to *Spinturnix bakeri*, another North American *Spinturnix* mite parasitizing bats, but can be distinguished by the number and placement of setae. *Spinturnix bakeri* possesses long posteroventral and anteroventral setae on leg II and leg III, respectively; the pair of proximal dorsal setae on femora I and II consist of one long and one short seta, and the proximal posterodorsal setae of femora III and IV are long (Rudnik 1960; Whitaker 1982). In comparison, *S. americanus* possesses tiny ventral setae and proximal dorsal setae of femora I and II, generally giving *S. americanus* a less hairy appearance than *S. bakeri* (Rudnik 1960; Whitaker 1982). The flea *Myodopsylla insignis* is a member of the Ischnopsyllidae family which exclusively parasitizes bats (Marshall 1982; Czenze and Broders 2011). *Myodopsylla insignis* is identified based on the presence of two genal spines on the front portion of the head instead of the back, a truncate maxilla, the presence of a pronotal comb, and the plate of the anterior portion of the head is wide and smooth—almost appearing translucent under some microscope lighting (Whitaker 1982). The eastern bat bug *Cimex adjunctus* is the largest ectoparasite collected from bats in Atlantic Canada and is similar in appearance to other *Cimex* species, including the bed bug *C. lectularius*. *Cimex adjunctus* is distinguished from other *Cimex* species in North America by the presence of long, thin bristles at the side of the pronotum that are only slightly serrated at the ends where other species have noticeably serrate bristles (Usinger 1966). The pronotal bristles of *C. adjunctus* are as long as or longer than the width of the eyes (Whitaker 1982).

### 2.3 Host–Parasite Associations

A matrix representing ectoparasite species abundance on both *Myotis lucifugus* and *M. septentrionalis* was generated for Atlantic Canada collectively and for each region. The associations between host and ectoparasites were visualized using the R package *circlize* (Gu et al. 2014).

### 2.4 Infection Parameters

Infection parameters of prevalence and mean intensity were calculated for each ectoparasite species as per Busht et al. (1997). Prevalence is the number of hosts infected with a given parasite species divided by the number of hosts examined and mean intensity is the sum of the number of parasites of a given species divided by the number of hosts infected (Busht et al. 1997). Prevalence and mean intensity were calculated for each host species as a whole and for age and sex demographics across regions in Atlantic Canada. Prevalence of each ectoparasite was compared to the number of hosts examined per region as an estimate of sampling effort to check for correlation (Bell and Burt 1991; Jovani and Tella 2006). Infection parameters and correlation between prevalence and number of hosts were calculated using the “cor.test” function with the Pearson correlation coefficient in R (R Core Team 2023).

### 2.5 Modeling Variation in Ectoparasite Load

After identifying the ectoparasites infecting *M. lucifugus* and *M. septentrionalis* in Atlantic Canada, we used generalized linear mixed models (GLMMs) to quantify the extent to which intra‐ and interspecific variation in ectoparasite load can be explained by differences between hosts. We focused on the two most common ectoparasites of these bats: the wing mite *Spinturnix americanus* and the bat flea *Myodopsylla insignis* . We separately investigated the factors affecting their prevalence and abundance on *M. lucifugus* and *M. septentrionalis* as these two ectoparasites exhibit different relationships with their host and have different life history strategies and environmental constraints. Collection data for *S. americanus* and *M. insignis* were converted to presence/absence data to test for factors affecting the presence of infection while counts of fleas or mites per host bat were used to examine factors affecting ectoparasite abundance.

We used GLMMs to assess whether the fixed effects of bat species, sex, and age class influenced mite and flea infections. GLMMs are parametric models suitable for ecological studies where response variables are counts and random effects are needed to account for variation within the data, such as the different collection sites and years included in this study (Bolker et al. 2009; O'Hara and Kotze 2010). To test for effects on ectoparasite presence, logistic regression models with a binomial distribution were used with presence as a binary response variable (1 = infected and 0 = uninfected) and the cloglog link function (Van Horn 2015; Zamora‐Mejías et al. 2020). To test for effects on ectoparasite abundance, logistic regression models with a Poisson distribution were used with abundance as the response variable and the log‐link function (O'Hara and Kotze 2010; Wood et al. 2020; Zamora‐Mejías et al. 2020).

All bats for which ectoparasite presence/absence, host species, age class, and sex data were available were used to test for effects on presence. A subset of the data, consisting of all bats with the above criteria that were caught and examined in 2010, was used to test for effects on abundance as more systematic sampling was conducted during this year (Czenze and Broders 2011) and abundance data from this year was considered exceptional based on Cook's distance results when diagnostics were done on the global models with the full data set. Highly sampled sites (Rawdon Mine, NS and Salmonier Nature Park, NFLD) were influential (Cook's distance > 1); however, this is due to greater numbers of bats being examined in these locations as part of other studies and this data was not removed. Ectoparasite collections were greatest in 2010 with 877 total ectoparasites collected, with 373 *Myodopsylla insignis* and 451 *Spinturnix americanus* collected.

The global model for presence included host species ( *M. lucifugus* or *M. septentrionalis* ), host sex (female or male), and host age class (adult or juvenile) as fixed effects and site and year as random effects:

$$\text{Presence}\sim \beta_{1}\left(\text{species}\right)+\beta_{2}\left(\mathrm{sex}\right)+\beta_{3}\left(\mathrm{age}\;\text{class}\right)+\left(1|\text{site}\right)+\left(1|\text{year}\right)$$

The global model for abundance included host species, host sex, and host age class as fixed effects and site as a random effect:

$$\text{Abundance}\sim \beta_{1}\left(\text{species}\right)+\beta_{2}\left(\mathrm{sex}\right)+\beta_{3}\left(\mathrm{age}\;\text{class}\right)+\left(1|\text{site}\right)$$

Candidate models investigated the effects of host species, sex, and age class separately to test for host effects. Host species is included to evaluate differences in infection between *Myotis lucifugus* and *M. septentrionalis* that may be a result of roosting or social differences at the species level (Lewis 1995; Menzel et al. 2002; Broders and Forbes 2004; Johnson et al. 2009). Previous work has found variation in infection between adult female, adult male, and young‐of‐the‐year for both species (Czenze and Broders 2011), as such age class and sex are included to account for this variation and test whether these patterns persist more widely for these host species in Atlantic Canada. To account for spatial variation in bat and ectoparasite distributions, site is included as a random effect. Year is included as a random effect to account for sampling variation across years for prevalence but not for abundance as only data from 2010 were included in the models for abundance. Seven *M. lucifugus* individuals had two ectoparasite collections within the same year of either *M. insignis, S. americanus*, or both, from which one record was chosen randomly to be included in the dataset. Individual was not included as a random effect in either the presence or abundance models to avoid singularities and overfitting (Barr et al. 2013).

GLMMs were fit using the ‘glmer’ function in the package *lme4* (Bates et al. 2015) in R (R Core Team 2023). Global and candidate models were assessed using AIC_c_ to account for small sample sizes (Burnham and Anderson 2002). Model ranking was done using the function ‘model.sel’ from the *MuMIn* package (Bartoń 2023). Models in the 95% confidence set were averaged using ‘model.avg’ from the package *MuMIn* (Burnham and Anderson 2002; Bartoń 2023) and the 95% confidence intervals of the b values were calculated using ‘confint’ (R Core Team 2023) on the model average, which reports values based on the conditional average.

### 2.6 Ethics Approval

All bats were captured and released according to animal care protocols approved by the animal care committees of Saint Mary's University, Halifax, Nova Scotia and the University of Waterloo, Waterloo Ontario. Wildlife scientific research permits were obtained from the government agencies of the provinces of Nova Scotia, New Brunswick, Prince Edward Island, and Newfoundland and Labrador.

## 3 Results

We collected and identified 2738 ectoparasites from 1088 *Myotis lucifugus* and 493 ectoparasites from 262 *Myotis septentrionalis* from 60 sites across Atlantic Canada between 1999 and 2017 (Figure 1). Included in this collection are 582, 28, and 60 specimens previously identified by Czenze and Broders (2011), Talbot et al. (2016), and Banerjee et al. (2020), respectively. Most specimens were collected from Newfoundland (1443) and Nova Scotia (1254) as part of long‐term studies in these regions. Fewer ectoparasites were collected from Labrador (368), New Brunswick (140), and Prince Edward Island (26). Four orders and five species were identified from the collection. An additional 29 ectoparasite specimens were also collected that were too small or damaged for species‐level identification.

### 3.1 Effects of Sampling

**FIGURE 2:**
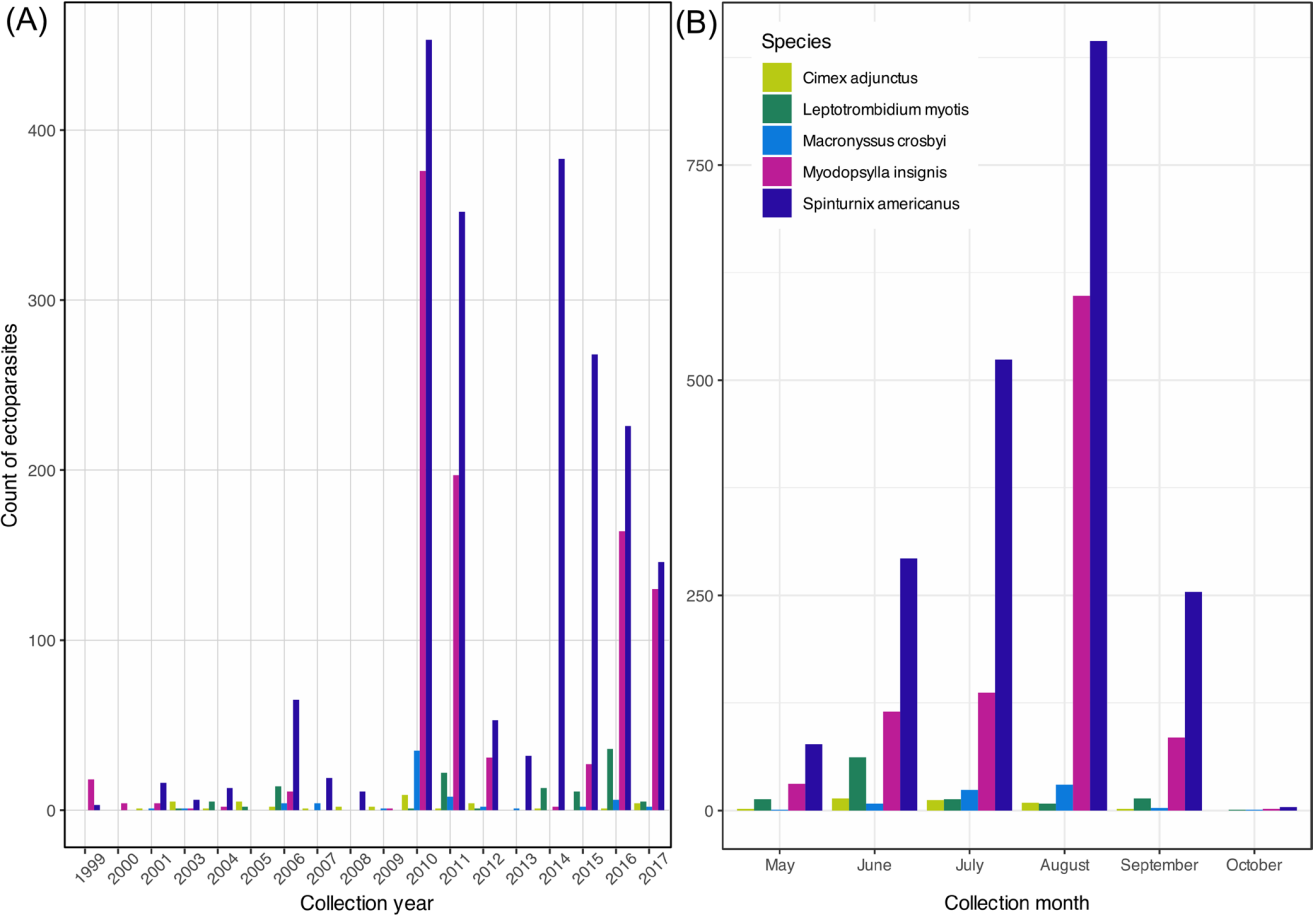
Counts of ectoparasites collected from Atlantic Canada by (A) year and (B) month. Peaks in ectoparasite collection over the years coincide with peak sampling years when more bat research was being conducted in Atlantic Canada (e.g., New Brunswick and Nova Scotia in 2010 and 2011 and Newfoundland in 2014–2017). Peaks in ectoparasite collections by month coincide with bat activity following parturition and during swarming and likely coincide with ectoparasite reproduction for some species.

The ectoparasites in this study were collected during research of the host bat species; as such peaks in ectoparasite collection over years coincide with years when more bat research was being conducted in Atlantic Canada (e.g., New Brunswick and Nova Scotia in 2010 and 2011 and Newfoundland in 2014–2017; Figure 2). As this research was carried out during months bats are active in Atlantic Canada, the peaks in ectoparasite collections by month coincide with bat activity, particularly between July and September following parturition and during autumn swarming. The prevalence of the ectoparasites is not significantly correlated with the number of hosts sampled for *M. lucifugus* (Table S1). Three *M. septentrionalis* ectoparasites (*S. americanus, C. adjunctus*, and *M. insignis* ) show no significant correlation between prevalence and the number of hosts sampled whereas the rarer *L. myotis* and *M. crosbyi* show significant positive correlations (≥ 0.98, *p* ≤ 0.02) between the prevalence of these ectoparasites and the number of hosts examined. The correlation between prevalence of the rare ectoparasites and the number of bats examined is expected as rarer species require more effort to detect such that rare ectoparasites may evade detection when it is not possible to examine all bats.

### 3.2 Host–Parasite Associations

**FIGURE 3:**
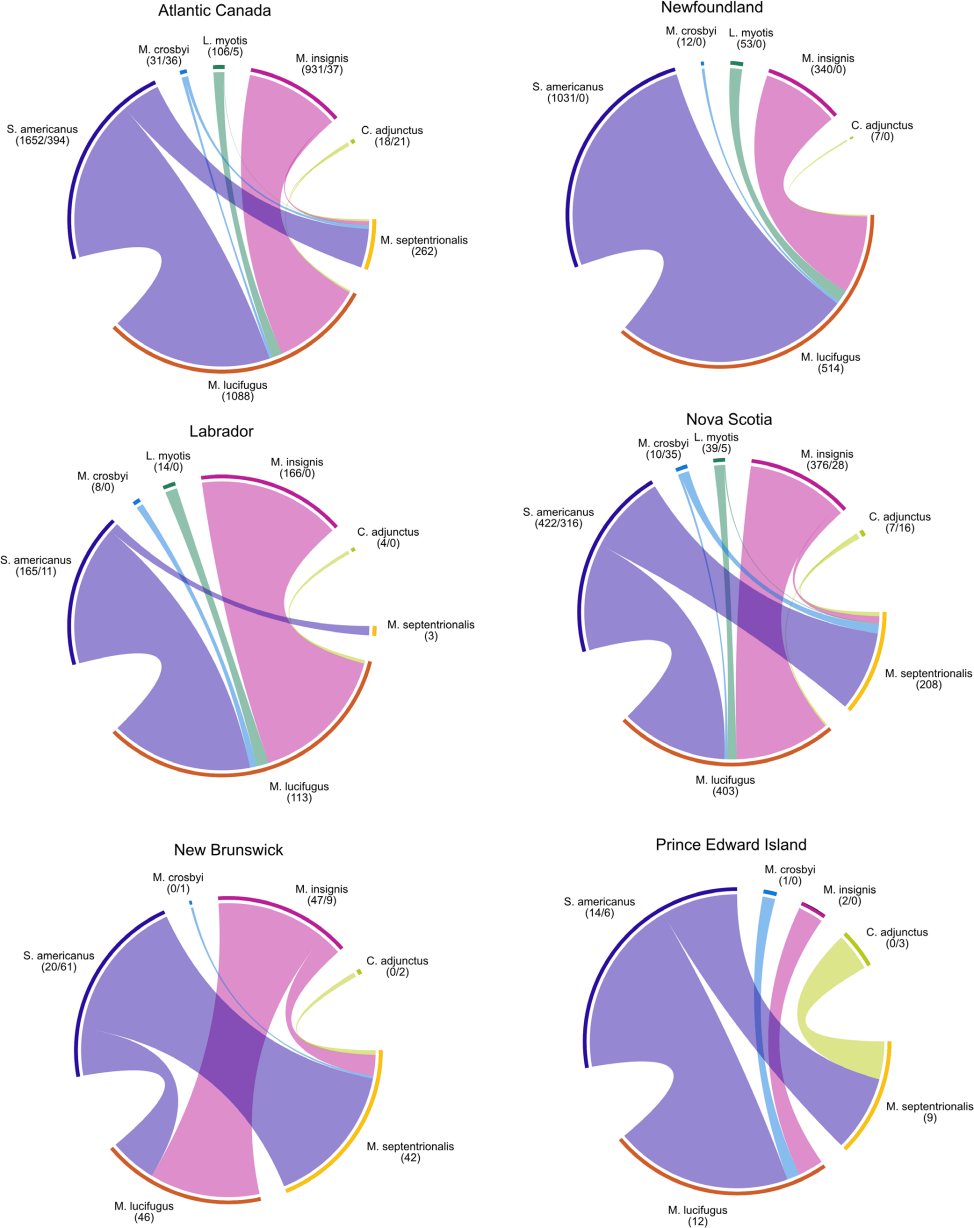
Host–parasite associations between *Myotis lucifugus* and *M. septentrionalis* and their ectoparasites in Atlantic Canada. The ectoparasite‐bat interactions within each region and Atlantic Canada as a whole are represented by a chord diagram. The curved bars illustrate the interactions between host and ectoparasite species with the size proportional to the number of interactions. The number of ectoparasites collected from each host is included in parentheses under the ectoparasite species names (number collected from *M. lucifugus* /number collected from *M. septentrionalis* ). The number of infected hosts from each region is included below the bat species names.

The ectoparasite assemblages of *Myotis lucifugus* and *M. septentrionalis* shared five ectoparasite species: *Spinturnix americanus, Myodopsylla insignis, Macronyssus crosbyi, Cimex adjunctus*, and *Leptotrombidium myotis* (Figure 3). The highest number of host–parasite interactions for both *M. lucifugus* and *M. septentrionalis* occurs with *S. americanus*, followed by *M. insignis* while infections with other ectoparasites appear to be rare during the collection period. This pattern is consistent across regions in Atlantic Canada.

### 3.3 Other Arthropod Collections

*Myotis septentrionalis* was also an incidental host to *Orchopeas caedens*, a squirrel flea, for which there was no evidence of its occurrence on *M. lucifugus* . *Androlaepas casalis*, a predatory mite, was also collected from both host species.

### 3.4 Infection Parameters

For *Myotis lucifugus*, the wing mite *Spinturnix americanus* and the flea *Myodopsylla insignis* exhibited consistently high prevalence of infection (41.2%–79.6% and 16.7%–73.9% for *S. americanus* and *M. insignis*, respectively) across regions while the prevalence of infection was below 10% for other ectoparasites (Table 1). *Spinturnix americanus* and *M. insignis* also showed average infection intensity greater than 1.0 parasite per infected host as did *Leptotrombidium myotis*. For *M. septentrionalis* , *S. americanus* exhibited the highest prevalence and average infection intensity with other ectoparasites either not being present or present at low numbers (Table 2). *Spinturnix americanus* and *M. insignis* were present on males and females of both adult and juvenile *M. lucifugus* and *M. septentrionalis* at varying levels of intensity (Tables S2–S5).

**TABLE 1:** Prevalence and mean intensity of ectoparasite infections on *Myotis lucifugus* for each region.

Ectoparasite	Labrador (113)		Newfoundland (514)		Nova Scotia (403)		New Brunswick (46)		Prince Edward Island (12)	
	P	I	P	I	P	I	P	I	P	I
CIAD	3.5 (4)	1.0 +/− 0.0	1.0 (5)	1.4 +/− 0.4	1.7 (7)	1.0 +/− 0.0	0	0	0	0
LEMY	6.2 (7)	2.0 +/− 1.0	1.9 (10)	5.3 +/− 2.5	4.5 (18)	2.2 +/− 0.8	0	0	0	0
MACR	6.2 (7)	1.1 +/− 0.1	1.9 (10)	1.2 +/− 0.1	2.0 (8)	1.3 +/− 1.3	0	0	8.3 (1)	1.0 +/− 0.0
MYIN	34.5 (39)	4.3 +/− 1.0	27.6 (142)	2.4 +/− 0.3	42.9 (173)	2.2 +/− 0.2	73.9 (34)	1.4 +/− 0.1	16.7 (2)	1.0 +/− 0.0
SPAM	70.8 (80)	2.1 +/− 0.2	79.6 (409)	2.5 +/− 0.1	60.8 (245)	1.7 +/− 0.1	41.2 (14)	1.4 +/− 0.2	75 (9)	0.6 +/− 0.2

*Note:* The number of bats sampled per region is included in brackets beside the region name. The number of infected individuals is included in brackets beside the prevalence values. *P* = prevalence of infection (%); I = mean intensity of infection (+/− SE).

Abbreviations: CIAD, *Cimex adjunctus* ; LEMY, *Leptotrombidium myotis*; MACR, *Macronyssus crosbyi*; MYIN, *Myodopsylla insignis* ; SPAM, *Spinturnix americanus*.

**TABLE 2:** Prevalence and mean intensity of ectoparasite infections on *Myotis septentrionalis* for each region.

Ectoparasite	Labrador (3)		Nova Scotia (208)		New Brunswick (42)		Prince Edward Island (9)	
	P	I	P	I	P	I	P	I
CIAD	0	0	6.7 (14)	1.1 +/− 0.1	4.8 (2)	1.0 +/− 0.0	33.3 (3)	1.0 +/− 0.0
LEMY	0	0	1.0 (2)	2.5 +/− 1.5	0	0	0	0
MACR	0	0	8.2 (17)	2.1 +/− 0.7	2.4 (1)	1.0 +/− 0.0	0	0
MYIN	0	0	13.0 (27)	1.0 +/− 0.0	21.4 (9)	1.0 +/− 0.0	0	0
SPAM	100 (3)	3.7 +/− 1.7	80.8 (168)	1.9 +/− 0.1	85.7 (36)	1.7 +/− 0.2	66.7 (6)	1.0 +/− 0.0

*Note:* The number of bats sampled per region is included in brackets beside the region name. The number of infected individuals is included in brackets beside the prevalence values. *P* = prevalence of infection (%); I = mean intensity of infection (+/− SE).

Abbreviations: CIAD, *Cimex adjunctus* ; LEMY, *Leptotrombidium myotis*; MACR, *Macronyssus crosbyi*; MYIN, *Myodopsylla insignis* ; SPAM, *Spinturnix americanus*.

### 3.5 Modeling Variation in Ectoparasite Load

**FIGURE 4:**
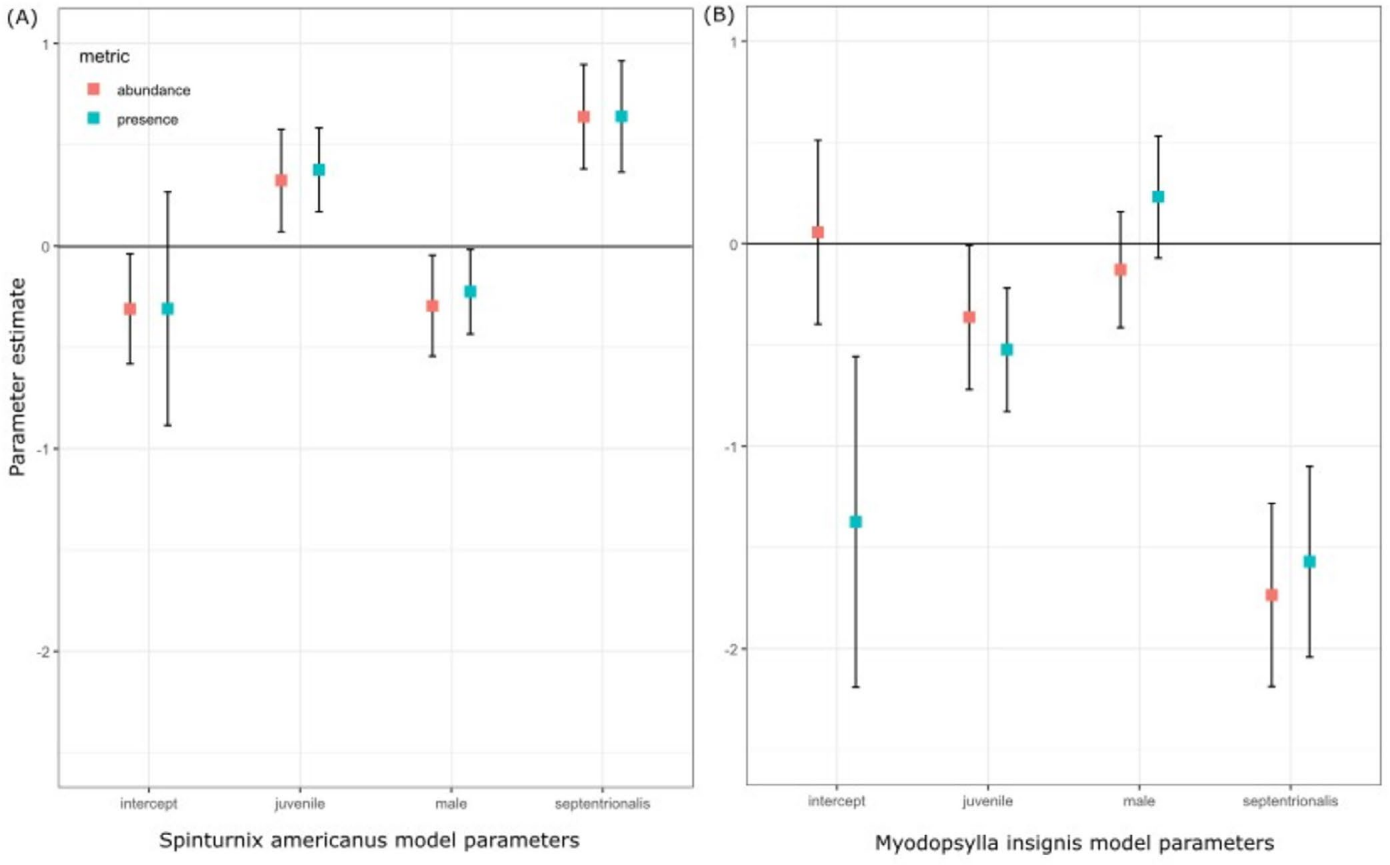
Model‐averaged parameter estimates and 95% confidence intervals for variables affecting presence and abundance of *S. americanus* (A) and *M. insignis* (B). Points represent the beta coefficients for the parameter estimates and the error bars represent the lower and upper limits of 95% confidence intervals. Confidence intervals that include zero (0) are considered non‐significant.

The modeling dataset included 980 *Myotis lucifugus* and 195 *M. septentrionalis*. Through model selection using AIC_c_ it was determined that the global model best explained mite presence and abundance (*w* = 0.77 and *w* = 0.82, respectively, Table 3). For the fleas, the global model best explained presence (*w* = 0.53) whereas the model that included host species and age class (model 1) best explained flea abundance (*w* = 0.54, Table 4). Model‐averaged parameter estimates were calculated for the models in the 95% confidence set and suggested that host species and host age class explained the most variation in mite and flea infection as the 95% CIs did not cross 0 (Figure 4). Variance for the random effect of year was high for the presence‐averaged models (variance = 1.67 ± 1.29 SD and variance = 0.923 ± 0.961 for *M. insignis* and *S. americanus* respectively), which is consistent with the different levels of sampling effort between years (Figure 2). Variance for site was < 1 for all averaged models, suggesting less variation in the collection of ectoparasites between sites.

**TABLE 3:** Models explaining the presence and abundance of *Spinturnix americanus* infections on *Myotis lucifugus* and *M. septentrionalis* in Atlantic Canada between 1999 and 2017.

Model		*K*	AIC_c_	∆AIC	*w*_i_	∑w_i_
Presence						
Global model	Age class + species + sex + (1|site) + (1|year)	7	1219.9	0	0.77	0.77
Model 1	Age class + species + (1|site) + (1|year)	6	1222.4	2.47	0.23	1.00
Model 2	Species + (1|site) + (1|year)	5	1231.3	11.35	0	
Model 3	Age class + (1|site) + (1|year)	5	1240.7	20.76	0	
Null	1 + (1|site) + (1|year)	5	1251.7	31.80	0	
Model 4	Sex + (1|site) + (1|year)	5	1253.6	33.68		
Abundance						
Global model	Age class + species + sex + (1|site)	5	749.9	0	0.82	0.82
Model 1	Age class + species + (1|site)	4	753.3	3.43	0.15	0.96
Model 2	Species + (1|site)	3	756.1	6.23	0.04	1.00
Model 3	Age class + (1|site)	3	774.0	24.16	0	
Model 4	Sex + (1|site)	3	778.9	29.01	0	
Null	1 + (1|site)	2	779.2	29.34	0	

*Note:* Models in the 95% confidence set are highlighted. *K* = number of parameters; AIC_c_ = Akaike information criterion for small sample sizes; *w*_i_ = Akaike weight; ∑*w*_i_ = sum of Akaike weights.

**TABLE 4:** Models explaining the presence and abundance of *Myodopsylla insignis* infections on *Myotis lucifugus* and *M. septentrionalis* in Atlantic Canada between 1999 and 2017.

Model		*K*	AIC_c_	∆AIC	*w*_i_	∑*w*_*i*_
Presence						
Global model	Age class + species + sex + (1|site) + (1|year)	7	1169.6	0	0.53	0.53
Model 1	Age class + species + (1|site) + (1|year)	6	1169.8	0.24	0.47	1.00
Model 2	Species + (1|site) + (1|year)	5	1179.6	10.05	0	
Model 3	Age class + (1|site) + (1|year)	5	1221.1	51.54	0	
Null	1 + (1|site) + (1|year)	5	1235.2	65.58	0	
Model 4	Sex + (1|site) + (1|year)	5	1237.0	67.43	0	
Abundance						
Model 1	Age class + species + (1|site)	4	647.4	0	0.54	0.54
Global model	Age class + species + sex + (1|site)	5	648.7	1.29	0.28	0.82
Model 2	Species + (1|site)	3	649.6	2.20	0.18	1.00
Model 3	Age class + (1|site)	3	723.7	76.30	0	
Null	1 + (1|site)	2	730.8	83.36	0	
Model 4	Sex + (1|site)	3	730.9	83.49	0	

*Note:* Models in the 95% confidence set are highlighted. *K* = number of parameters; AIC_c_ = Akaike information criterion for small sample sizes; *w*_i_ = Akaike weight; ∑*w*_i_ = sum of Akaike weights.

The effects of host demographics on the abundance and presence of the two ectoparasites varied between the ectoparasites (Figure 4). *Spinturnix americanus* was more likely to infect *M. septentrionalis* than *M. lucifugus*, with more abundant infections on *M. septentrionalis* . In contrast, *M. insignis* was less likely to be present on *M. septentrionalis* than *M. lucifugus*, with lower abundance. For age demographics, *S. americanus* was more likely to be present on juveniles relative to adults, whereas *M. insignis* was less likely to be present on juveniles. Sex was a significant determinant of infection for *S. americanus*, with males exhibiting a weak negative relationship relative to females, with males being less likely to be infected than females. Sex was not a significant parameter for *M. insignis* as the 95% CI crossed 0 for flea prevalence and infection.

## 4 Discussion

The bat ectoparasite assemblages in Atlantic Canada consisted of five species for both *M. lucifugus* and *M. septentrionalis*. Assemblages for both were dominated by mites (Mesostigmata) and fleas (Siphonaptera), particularly the wing mite *Spinturnix americanus* and the bat flea *Myodopsylla insignis* . Parasites often exhibit aggregated distributions (Poulin 2007), and this is especially evident in the rare ectoparasites of *M. lucifugus* and *M. septentrionalis* , particularly for the chigger mite *Leptotrombidium myotis* which was abundant on a select number of bats. The ectoparasite assemblages found across Atlantic Canada were consistent with those previously found at swarming sites (Poissant and Broders 2008; Czenze and Broders 2011). These assemblages were more species‐rich than assemblages on *M. lucifugus* in Manitoba for which there was only evidence of infection with *S. americanus* and *M. insignis* (Webber, McGuire, et al. 2015) but lower than that seen for other bat species in the central U.S.A. (Pearce and O'Shea 2007; Schumann 2019). Differences in ectoparasite assemblages across North America may be related to several factors, including temperature and humidity tolerances of the ectoparasites (Benoit and Denlinger 2010; Mize et al. 2011) and the diversity or density of host species in an area (Dick and Patterson 2007; Kamiya et al. 2014). The family‐level ectoparasite assemblage composition in Atlantic Canada is similar to temperate bats in Europe; however, European bats can also be infected with ticks (Argasidae and Ixodidae) and bat flies (Nycteribiidae) (Zahn and Rupp 2004; McKee et al. 2016; Sándor et al. 2019).

The presence and abundance patterns of the two most common ectoparasites, *Myodopsylla insignis* and *Spinturnix americanus*, arise from a combination of ectoparasite and bat life history traits. *Myodopsylla insignis* was found on both bat species at summer and swarming sites, suggesting multiple generations are occurring on both species throughout the year; however, it was found at higher prevalence and intensity on *M. lucifugus* than on *M. septentrionalis*. Our analysis found that *M. septentrionalis* was less likely to be infected by fleas than *M. lucifugus* and supports previous reports of *M. lucifugus* as the more compatible host (Smith and Clay 1988), likely due to the differences in roosting behavior between the two bat species. Like other flea species, *M. insignis* is oviparous with a high reproductive out put (Marshall 1982). Adult, female *M. insignis* likely produce many eggs a day, laying potentially hundreds in a lifetime (Marshall 1982). Early life stages of *M. insignis* develop within the roost litter and require bats to continue to use the roost for newly emerged adult fleas to find new hosts (Smith and Clay 1988). This flea life history strategy may be more compatible with *M. lucifugus* as they congregate in larger groups and switch roosts less frequently than *M. septentrionalis* (Lewis 1995; Menzel et al. 2002; Broders and Forbes 2004; Johnson et al. 2009). The differences in social behavior may mean that *M. lucifugus* is a more stable and reliable food source for *M. insignis* , allowing for the maintenance of larger populations of fleas whereas the more frequent roost switching of *M. septentrionalis* results in a lower probability of being able to continue their life cycle. Additionally, the roosting locations of *M. lucifugus* may have more favorable substrate or microclimate conditions for the early stages of *M. insignis* development (Smith and Clay 1988).

In comparison, *S. americanus* mites are permanent ectoparasites that complete their life cycle attached to the membranes of their host and require direct contact with potential new hosts for transmission (Rudnik 1960; Christe et al. 2000; Ter Hofstede and Fenton 2005). *Spinturnix americanus*, like other spinturnid mites, is viviparous where female spinturnid mites embryonate eggs and larva internally before giving birth to the blood‐feeding protonymph (Orlova et al. 2018). This reproductive method ensures the next generation can parasitize a host immediately; however, it does result in a lower reproductive output than *M. insignis* . The lower reproductive capacity of *S. americanus* coupled with their reduced mobility may reduce their ability to infect all members of large colonies. *Spinturnix americanus* was found at greater than 60% prevalence for both host species in all regions, except New Brunswick for *M. lucifugus*, and the modeling results suggest that these mites are more likely to be infecting *M. septentrionalis* than *M. lucifugus* . The life cycle and direct transmission of *S. americanus* may enable the mites to persist regardless of host roosting behavior, unlike *M. insignis* . The higher density of bats at *M. lucifugus* roosts may allow mites to preferentially aggregate on the most susceptible batsrather than spreading to the majority of bats in the smaller roost groups of *M. septentrionalis* . Further analysis of the phenology of *S. americanus* infections on *M. lucifugus* in correlation with host age and reproductive status (e.g., pregnant, lactating, etc.) could provide insight into the possibility of which bats are the more susceptible or preferred hosts. *Spinturnix* mites are considered host‐specific ectoparasites that feed on one or a few closely related host species (Bruyndonckx et al. 2009) and *S. americanus* has been found parasitizing several North American *Myotis* bats (Whitaker and Wilson 1974), suggesting that they may not have a particular preference for *M. lucifugus* or *M. septentrionalis* , but for *Myotis* bats generally. The presence of *S. americanus* on both *M. lucifugus* and *M. septentrionalis* across Atlantic Canada is consistent with previous investigations in Nova Scotia and New Brunswick (Poissant and Broders 2008; Czenze and Broders 2011) and confirms that *S. americanus* is a widespread ectoparasite of *Myotis* bats in Atlantic Canada.

*Spinturnix americanus* and *M. insignis* also appear to have different infection patterns related to the age and sex of hosts. *Spinturnix americanus* was more likely to infect juvenile bats whereas *M. insignis* was more likely to infect adult bats. Mother bats and their offspring are in close proximity while bats are young, especially during the lactation period, and this may facilitate transmission of mites to juvenile bats (Christe et al. 2000; Ter Hofstede and Fenton 2005). The limited mobility of *S. americanus* would suggest that they were likely feeding on the juvenile bats they were collected from. While our results suggest *M. insignis* was found more often on adult bats, *M. insignis* has high mobility between individuals within the roost relative to *S. americanus* and the bats were only captured outside the roost. As such, we cannot confirm whether the fleas preferentially feed on adult bats over juveniles when bats are roosting. Previous work by Czenze and Broders (2011) found a higher prevalence of ectoparasites on adult females than males of *M. lucifugus* and *M. septentrionalis* , particularly for *S. americanus* infections which is consistent with our wider study. There was no relationship to host sex found for *M. insignis* in our study and this may be due to no preference by the ectoparasite or a limitation of the data as fewer bats were infected with *M. insignis*.

The rare ectoparasite fauna of *M. lucifugus* and *M. septentrionalis* consists of *Cimex adjunctus, Leptotrombidium myotis*, and *Macronyssus crosbyi*. Each of these ectoparasites was previously identified from *M. lucifugus* and *M. septentrionalis* by Czenze and Broders (2011) in Nova Scotia and New Brunswick and *C. adjunctus* was identified from Prince Edward Island and Newfoundland and Labrador by Talbot et al. (2016). *Cimex adjunctus* and *L. myotis* are both temporary ectoparasites of bats. While *C. adjunctus* is widespread in North America east of the Rocky Mountains (Usinger 1966; Czenze and Broders 2011; Bowles et al. 2013; Talbot et al. 2016), it is only present on the bats to feed and spends much of its time within the roost. Bats were checked for ectoparasites during netting events at maternity colonies or in flyways and this may have reduced the likelihood of collecting bat bugs that only occasionally disperse with their hosts (Usinger 1966). *Leptotrombidium myotis* is parasitic in its larval stage and free‐living as adults (Whitaker 1982). Trombiculid larvae may be able to feed on mammal species other than their primary host but are often restricted to the available hosts in the habitat of adult Trombiculids (Whitaker 1982; Schumann 2019). *Leptotrombidium myotis* was collected from the ears of *M. lucifugus* and *M. septentrionalis* , as is typical for this species (Pearce and O'Shea 2007; Czenze and Broders 2011). The presence of *L. myotis* on either bat species may be more dependent on environmental factors influencing its other life stages. *Macronyssus crosbyi* has previously been identified from *E. fuscus* and several *Myotis* bats throughout North America (Whitaker and Wilson 1974). Protonymphs of Macronyssid mites are often found crawling on the wings of their bat hosts which is consistent with our collection of *M. crosbyi* individuals from the wings of *M. lucifugus* and *M. septentrionalis* (Whitaker 1982). Other species of *Macronyssus* mites in the Palearctic have been found in greater abundance in autumn and winter (Estrada‐Peña and Serra‐Cobo 1991; Zhang et al. 2010) which suggests that *M. crosbyi* may reach greater abundances on *M. lucifugus* and *M. septentrionalis* during the later periods of swarming or during hibernation for these bat species.

Our collection also included two species that are unlikely to be direct ectoparasites of these bat species: the predatory mite *Androlaelaps casalis* and the squirrel flea *Orchopeas caedens. Androlaelaps casalis* is a predatory mite that feeds on eggs and larval stages of other mites, such as those parasitizing bats and bird nests as well as pests of cereal crops (Barker 1968; Whitaker and Wilson 1974). *Androlaelaps casalis* is likely incidental on little brown and northern myotis bats but is a potential predator of *L. myotis* and *M. crosbyi* when present. Similarly, *O. caedens* is likely incidental on *M. septentrionalis* as these bats commonly roost in trees and transmission could occur from the primary squirrel host. *Orchopeas caedens* was previously recorded from squirrels in New Brunswick and in Maine, U.S.A., from the northern flying squirrel ( *Glaucomys sabrinus* ; Brown 1955; Eckerlin and Gardner 2021).

This historical collection of ectoparasites provides us with a look into past relationships between *M. lucifugus* and *M. septentrionalis* and their arthropod ectoparasites prior to the arrival of the white‐nose syndrome (WNS) causing fungus *Pseudogymnoascus destructans* in Atlantic Canada. The fungus was first detected in the United States in 2006 and Canada in 2010 and since then most of the known hibernacula in the northeastern United States and eastern Canada have experienced catastrophic declines (Environment Canada 2018). Three bat species in Atlantic Canada have been declared endangered as a result of WNS and other factors: the little brown myotis ( *M. lucifugus* ), the northern myotis ( *M. septentrionalis* ), and the tri‐colored bat ( *Perimyotis subflavus* ; Environment Canada 2018). The declines of these host species can have repercussions for the survival of their ectoparasites as they can experience secondary extirpations when their hosts are removed from the ecosystem (Lafferty 2012; Wood et al. 2020). Given the host specificity of some of these ectoparasites, it is likely that they also experienced population declines alongside their hosts. Which ectoparasite species may still be persisting in remanent host populations is currently unknown.

Our work highlights the influence of life history traits and behaviors of both the ectoparasites and the hosts on the host–parasite interactions. The effect of life history traits on host–parasite relationships is apparent with *M. septentrionalis* exhibiting lower infections of *M. insignis* , potentially due to the temporary nature of the flea parasitism and the increased roost switching of *M. septentrionalis* . The permanent nature of the life history of *S. americanus* made it an almost ubiquitous parasite of examined hosts. Distributions across individual bats appear to be highly aggregated for the rare species. The secondary effects of the spread of *Pseudogymnoascus destructans* on the bat ectoparasites are currently unknown and highlight the importance of studying the parasites of focal species to understand the complexity of their interactions and create a more complete picture of an area's biodiversity. Future work on remaining or recovering bat populations should include investigations of the ectoparasites to determine if the communities have changed or persisted post‐WNS. Habitat management plans should take into consideration the need for bats to switch roosts to avoid high ectoparasite loads. For *M. lucifugus* this could include the provision of alternate roosts, such as bat boxes or rocket boxes, in locations with few maternity roost options whereas for *M. septentrionalis* the protection of mature trees, including standing dead or dying trees, throughout forested areas could provide potential suitable alternate roosts.

## Author Contributions

**Alexandra H. Sauk:** conceptualization (equal), data curation (lead), formal analysis (lead), investigation (equal), methodology (equal), visualization (equal), writing – original draft (lead), writing – review and editing (equal). **Hugh G. Broders:** conceptualization (equal), data curation (supporting), formal analysis (supporting), funding acquisition (lead), investigation (equal), methodology (equal), project administration (lead), resources (lead), supervision (lead), visualization (equal), writing – original draft (supporting), writing – review and editing (equal).

## Funding

This work was supported by the Natural Sciences and Engineering Research Council of Canada, 04743.

## Conflicts of Interest

The authors declare no conflicts of interest.

## Supporting information


**Table S1:** Correlation coefficients of prevalence of ectoparasites versus the number of sampled hosts.
**Table S2:** Prevalence of ectoparasite infections (%) on *Myotis lucifugus* for each region and demographic. Number of bats sampled per region is included in brackets beside the region name and the number of infected individuals is included in brackets beside the prevalence values.
**Table S3:** Mean intensity of ectoparasite infections on *Myotis lucifugus* for each region and demographic. Number of bats sampled per region is included in brackets beside the region name.
**Table S4:** Prevalence of ectoparasite infections (%) on *Myotis septentrionalis* for each region and demographic. Number of bats sampled per region is included in brackets beside the region name and the number of infected individuals is included in brackets beside the prevalence values.
**Table S5:** Mean intensity of ectoparasite infections on Myotis septentrionalis for each region and demographic. Number of bats sampled per region is included in brackets beside the region name.

## Data Availability

Specimen collection metadata and the subset of the data used for the infection modeling is archived on Dryad (DOI: https://doi.org/10.5061/dryad.h9w0vt4v3).
